# An Evaluation of the Predictive Value of Sepsis Patient Evaluation in the Emergency Department (SPEED) Score in Estimating 28-Day Mortality Among Patients With Sepsis Presenting to the Emergency Department: A Prospective Observational Study

**DOI:** 10.7759/cureus.22598

**Published:** 2022-02-25

**Authors:** Takshak Shankar, Nidhi Kaeley, Vempalli Nagasubramanyam, Yogesh Bahurupi, Archana Bairwa, D J L Infimate, Reshma Asokan, Krishna Shukla, Santosh S Galagali

**Affiliations:** 1 Emergency Medicine, All India Institute of Medical Sciences, Rishikesh, Rishikesh, IND; 2 Community & Family Medicine, All India Institute of Medical Sciences, Rishikesh, Rishikesh, IND

**Keywords:** 28-day mortality, piro score, meds score, speed score, sepsis

## Abstract

Background and objective

Sepsis is a life-threatening medical emergency and a significant cause of mortality. Risk stratification scores for sepsis can be unsuitable for use in the emergency department (ED) due to their complexity, and an appropriate solution has yet to be found. In this study, the predictive value of the Sepsis Patient Evaluation in the Emergency Department (SPEED) score in estimating 28-day mortality was assessed among patients with sepsis presenting to the ED, in order to determine its suitability as an efficient risk stratification system.

Materials and methods

This was a single-center, prospective observational study conducted at an urban tertiary care center. We included patients presenting to the ED with suspected or confirmed sepsis who met the inclusion and exclusion criteria of our study. The patients were evaluated with the following scoring systems on arrival: the SPEED score; Predisposition, Infection, Response, and Organ dysfunction (PIRO) score; and Mortality in Emergency Department Sepsis (MEDS) score; the patients were subsequently followed up on the 28th day to record the final outcomes with regard to mortality and discharge rates.

Results

This study included 127 patients in total. The median age of the study population was 49 years, and the 28-day mortality rate was 50.4%. The area under the receiver operating characteristic (AUROC) curve for the SPEED score for predicting mortality was 0.899 (95% CI: 0.847-0.951). In comparison, the AUROC for MEDS and PIRO scores was 0.857 (95% CI: 0.793-0.92) and 0.895 (95% CI: 0.838-0.951), respectively. Based on the DeLong test, no significant difference was found in the diagnostic performances with respect to these scores.

Conclusion

The SPEED score is a simple and handy parameter that can be used for the early and appropriate risk stratification of patients with sepsis in the ED.

## Introduction

Despite the advances in modern antibiotics and resuscitation measures, sepsis remains a major cause of morbidity and mortality worldwide. In 2017, there were an estimated 48.9 million cases of sepsis. Globally, there were 11 million sepsis-related deaths, representing around 19.7% of all deaths worldwide [1]. In India, patients with sepsis and septic shock have a reported mortality rate of 35.8% and 63.6%, respectively, making sepsis a significant burden on the Indian healthcare system [2].

According to the SEPSIS-3 guidelines of 2016, sepsis is a life-threatening organ dysfunction caused by the dysregulated, overactive host response to an infection. An increase in the Sequential Organ Failure Assessment (SOFA) score of ≥2 points qualifies as organ dysfunction. Septic shock is a subtype of sepsis, where the underlying circulatory and cellular/metabolic abnormalities are profound enough to significantly increase mortality. Clinically, it is diagnosed by the presence of sepsis and the requirement of vasopressor therapy to elevate MAP (Mean Arterial Pressure) ≥65 mm/Hg and lactate levels >2 mmol/L (18 mg/dL) despite adequate fluid resuscitation [3].

In order to reduce mortality related to sepsis, early detection and aggressive treatment of patients are essential [4,5]. Patients with sepsis usually present to the emergency department (ED) initially for evaluation. While these patients may not appear too ill at initial presentation, their condition can deteriorate rapidly. Accurate assessment of the severity and risk for mortality at initial presentation becomes essential in the ED, to differentiate patients who require intensive care from patients who can be managed in the wards. Given the typical time constraints in the ED, these decisions about patient management and disposition need to be made quickly and efficiently. Patients who are transferred to an ICU directly from the ED have lower mortality rates than those who need to be shifted to an ICU from the regular wards [6,7]. Thus, appropriate and timely classification of patients in the ED would aid in the proper allocation of healthcare resources, as well as significantly reduce patient morbidity and mortality.

Sepsis patients can be classified based on various methods, including clinical judgment, scoring systems, and sepsis categories as defined by the Surviving Sepsis Campaign [5]. However, stratification based on scoring systems and clinical judgment has been shown to be superior to classification based on sepsis categories [8]. There are various scoring systems for patients with sepsis that aid not only in determining the severity but also in predicting mortality. These include the Acute Physiology and Chronic Health Evaluation (APACHE) score; Simplified Acute Physiology Score (SAPS); Predisposition, Infection, Response, and Organ dysfunction (PIRO) score; Mortality in Emergency Department Sepsis (MEDS) score; Rapid Emergency Medicine Score; Mortality In Severe Sepsis in the Emergency Department (MISSED) score; Sequential Organ Failure Assessment (SOFA) score; quick SOFA (qSOFA) score; and the Sepsis Patient Evaluation in the Emergency Department (SPEED) score [3,9-17].

Some of these scoring systems are quite comprehensive and were originally designed for usage in the ICU, such as the APACHE score. They require information that may not be readily available to an ED physician, which limits their utility in that setting. Certain scoring systems have been developed for this very purpose in recent years, to guide ED physicians in clinical decision-making and appropriate disposition of patients with sepsis. These scoring systems are simple to calculate, as they utilize limited parameters, yet they can accurately predict mortality. The MEDS score is one of the most widely used scoring systems in the ED. The PIRO score is also a comprehensive tool, and it was devised for usage in the ED. However, EDs in developing countries often have limited infrastructure and budget, and facilities for covariates like band cell count and differential blood cell counts may not be readily available everywhere. The SPEED score utilizes the most fundamental and readily available diagnostics and is simpler than both the MEDS and PIRO scores, making it very useful in the ED setting.

There have been very few studies on the SPEED score in the literature. In light of this, we conducted this study to assess the predictive value of the SPEED score in estimating the 28-day mortality among septic patients who present to the ED. We also compared the predictive value of the SPEED, MEDS, and PIRO scores in estimating the 28-day mortality of sepsis patients presenting to the ED.

## Materials and methods

This prospective observational study was conducted in the Department of Emergency Medicine at an urban tertiary care hospital. The purpose of this study was to assess the predictive value of the SPEED score for estimating 28-day mortality among patients with sepsis presenting to the ED, as well as compare it to the predictive values of the MEDS and PIRO scores.

We included adult patients who were 18 years of age or older who presented to the ED with suspected or confirmed sepsis and who fulfilled two or more criteria of the qSOFA score in the study. Pregnant females and patients who presented to the ED in cardiac arrest were excluded. Informed written consent was obtained from the patients or their relatives. The study was approved by the Institutional Ethical Committee at AIIMS, Rishikesh (Ref No: AIIMS/IEC/20/381).

Sample size

On review of the literature, the expected AUC of the SPEED, MEDS, and PIRO scores were >0.8, ~0.75, and ~0.9, respectively for estimating mortality among patients with sepsis who presented to the ED. The sample size calculated for each parameter at a corrected alpha of 0.017 and a conservative expected AUC of 0.75 was 71 (after adjusting for an attrition rate of 20% due to non-responders) [12,13,17]. A total of 127 patients were included in the study. The calculation was done by package pROC of the R statistical environment [18].

One ROC curve power calculation:

Ncases = 17.49195

Ncontrols = 52.47586

AUC = 0.75

Sig. level = 0.016

Power = 0.8.

Methods

Based on the inclusion and exclusion criteria, patients presenting to the ED with suspected or confirmed sepsis were screened for eligibility. If a patient was found to have met the eligibility criteria and once they gave informed written consent, their SPEED, MEDS, and PIRO scores were calculated. Table 1 describes the details related to this. Patients were grouped into four categories based on their SPEED score: <3 points, 4-6 points, 7-9 points, and >10 points [17]. Patients were also grouped into five categories according to their MEDS score: 0-4 points, 5-7 points, 8-11 points, 12-14 points, and ≥15 points [13]. Patients were additionally grouped into four categories according to their PIRO score: <5 points, 5-9 points, 10-14 points, and 15-19 points [12]. Each patient or their relatives were followed up on day 28, either through hospital records or the telephone, to record final outcomes in terms of survival or mortality.

**Table 1:** Parameters of the SPEED, MEDS, and PIRO scores SPEED: Sepsis Patient Evaluation in the Emergency Department; MEDS: Mortality in Emergency Department Sepsis; PIRO: Predisposition, Infection, Response, and Organ Dysfunction; COPD: chronic obstructive pulmonary disease; CLD: chronic liver disease; CNS: central nervous system; UTI: urinary tract infection; LRTI: lower respiratory tract infection; PaO_2_: partial pressure of oxygen; FiO_2_: fraction of inspired oxygen; aPTT: activated partial thromboplastin time; GCS: Glasgow Coma Scale

Parameters	Points
SPEED score	
Immunosuppressed state	3
Hypotension (systolic blood pressure <90 mmHg)	3
Hypothermia (body temperature <36 °C)	3
Hypoxemia (pulse oximetry <90%)	2
Low hematocrit (<0.38)	2
Elevated blood lactate (>2.4 mmol/L)	2
Pneumonia	2
Low pH (<7.35)	1
MEDS score	
Terminal illness (<30 days)	6
Hypoxia or tachypnea	3
Septic shock	3
Platelet count <150,000	3
Granulocytic bands >5%	3
Age >65 years	3
LRTI	2
Nursing home resident	2
Altered mental status	2
PIRO score	
Predisposition group	
Age >70 years	2
COPD	2
CLD	2
Cancer	3
Presence of Foley catheter	2
Infection group	
Pneumonia	1
CNS: meningitis/encephalitis	2
Abdomen: UTI	2
Response group	
Bands >5%	2
Respiratory rate >20/minute	3
Organ dysfunction group	
Systolic blood pressure <90 mmHg	1
PaO_2_/FiO_2_ <300	2
Urine output in the first 2 hours <30 mL	2
aPTT >35 seconds	1
Creatinine >1.8 mg/dL	1
GCS ≤9	3

Statistical analysis

The SPSS statistics software version 25 (IBM, Armonk, NY) was used to analyze the data [19]. Categorical variables were represented with numbers and percentages, and continuous variables were presented as mean (SD) and median (IQR) (depending on the distribution of the data after assessing normality via the Shapiro-Wilk test). A chi-squared test or Fisher’s exact test was used to analyze the categorical variables. Continuous variables were analyzed with the Wilcoxon-Mann-Whitney U test or a t-test. The receiver operating characteristic (ROC) curves were plotted for each of the three scores, and the area under the ROC curves (AUROC) was computed and analyzed. The level of significance was set at p<0.05.

## Results

After accounting for the inclusion and exclusion criteria, informed consent, and attrition, a total of 127 patients were included in this study. The study population was in the age group of 20-78 years, with a median (IQR) age of 49 (35-63) years. About half of the study population had at least one comorbidity, among which diabetes mellitus (29; 22.8%), hypertension (20; 15.7%), and malignancy (10; 7.9%) were the most common comorbidities; 84 patients (66.1%) required intubation and 90 patients (70.9%) were admitted to the ICU. By the end of 28 days, 64 patients (50.4%) had expired. The baseline characteristics of the patients in the study population are summarized in Table 2.

**Table 2:** Demographic, clinical parameters, and etiologies of all patients in the study population (n = 127) IQR: interquartile range; SD: standard deviation; BPM: beats per minute; CPM: counts per minute; GCS: Glasgow Coma Scale; HDU: high-dependency unit; ICU: intensive care unit

Variable	Values
Age, years, median (IQR)	49.00 (35.00–63.00)
Gender, n (%)	
Male	84 (66.1%)
Female	43 (33.9%)
Substance use, n (%)	
None	82 (64.6%)
Smoking	13 (10.2%)
Alcohol	3 (2.4%)
Tobacco	3 (2.4%)
Comorbidities, n (%)	
None	67 (52.8%)
Diabetes mellitus	29 (22.8%)
Hypertension	20 (15.7%)
Malignancy	10 (7.9%)
Chronic liver disease	7 (5.5%)
Chronic kidney disease	7 (5.5%)
Chronic obstructive pulmonary disease	4 (3.1%)
Hypothyroidism	4 (3.1%)
Coronary artery disease	3 (2.4%)
Stroke	3 (2.4%)
Bronchial asthma	2 (1.6%)
Pulmonary tuberculosis	2 (1.6%)
Etiology, n (%)	
Pneumonia	74 (58.3%)
Urinary tract	40 (31.5%)
Blood	14 (11%)
Meningitis	8 (6.3%)
Skin and soft tissue	4 (3.1%)
Intra-abdominal	1 (0.78%)
General physical examination	
Heart rate, BPM, mean ± SD	107.89 ± 9.64
Systolic blood pressure, mmHg, median (IQR)	96.00 (80.00–106.00)
Mean arterial pressure, mmHg, median (IQR)	76.00 (65.00–82.00)
Diastolic blood pressure, mmHg, median (IQR)	66.00 (58.00–70.00)
Respiratory rate, CPM, mmHg, median (IQR)	31.00 (24.00–36.00)
GCS, median (IQR)	15.00 (10.00–15.00)
Outcomes, n (%)	
Intubation	84 (66.1%)
ICU admission	90 (70.9%)
HDU admission	37 (29.1%)
Deceased	64 (50.4%)
Alive	63 (49.6%)

Table 3 summarizes the distribution of the SPEED, MEDS, and PIRO scores in the study population. The median SPEED, MEDS, and PIRO scores of the study population were 6, 8, and 10, respectively.

**Table 3:** Distribution of SPEED, MEDS, and PIRO scores in the study population (n = 127) SPEED: Sepsis Patient Evaluation in the Emergency Department; MEDS: Mortality in Emergency Department Sepsis; PIRO: Predisposition, Infection, Response, and Organ Dysfunction; COPD: chronic obstructive pulmonary disease; CLD: chronic liver disease; UTI: urinary tract infection; RR: respiratory rate; SBP: systolic blood pressure; LRTI: lower respiratory tract infection; PaO_2_: partial pressure of oxygen; FiO_2_: fraction of inspired oxygen; aPTT: activated partial thromboplastin time; GCS: Glasgow Coma Scale; IQR: interquartile range

Parameters	Values
SPEED score	
Hypoxemia (pulse oximetry <90%), n (%)	79 (62.2%)
Pneumonia, n (%)	75 (59.1%)
Low hematocrit (<0.38), n (%)	67 (52.8%)
Low pH (<7.35), n (%)	64 (50.4%)
Elevated blood lactate (>2.4 mmol/L), n (%)	61 (48.0%)
Hypotension (SBP <90 mmHg), n (%)	50 (39.4%)
Immunosuppressed state, n (%)	12 (9.4%)
Hypothermia (body temperature <36 °C), n (%)	7 (5.5%)
Total score, median (IQR)	6 (4–9)
MEDS score	
Hypoxia or tachypnea, n (%)	103 (81.1%)
LRTI, n (%)	74 (58.3%)
Granulocytic bands, n (%)	59 (46.5%)
Platelet count, n (%)	55 (43.3%)
Altered mental status, n (%)	51 (40.2%)
Septic shock, n (%)	50 (39.4%)
Age >65 years, n (%)	26 (20.5%)
Terminal illness, n (%)	1 (0.8%)
Nursing home resident, n (%)	0 (0.0%)
Total score, median (IQR)	8 (5–11)
PIRO score	
Foley catheter, n (%)	75 (59.1%)
Age >70 years, n (%)	18 (14.2%)
Cancer, n (%)	12 (9.4%)
COPD, n (%)	4 (3.1%)
CLD, n (%)	7 (5.5%)
Pneumonia, n (%)	75 (59.1%)
UTI, n (%)	40 (31.5%)
Meningitis/encephalitis, n (%)	9 (7.1%)
RR >20/minute, n (%)	103 (81.1%)
Bands >5%, n (%)	60 (47.2%)
Creatinine >1.8 mg/dL, n (%)	53 (41.7%)
SBP <90 mmHg, n (%)	50 (39.4%)
Urine output in the first 2 hours <30 mL, n (%)	50 (39.4%)
PaO_2_/FiO_2 _<300, n (%)	49 (38.6%)
aPTT >35 seconds, n (%)	16 (12.6%)
GCS ≤9, n (%)	28 (22.0%)
Total score, median (IQR)	10 (7–13)

Table 4 presents a comparison of the SPEED, MEDS, and PIRO scores between survivors and non-survivors. The median SPEED, MEDS, and PIRO scores were higher among non-survivors when compared to survivors. There was a significant difference between the two groups in terms of SPEED score (W = 408.500, p <0.001), MEDS score (W = 578.000, p <0.001), and PIRO score (W = 424.500, p <0.001).

Using point-biserial correlation, the strength of association between the SPEED score and mortality was 0.67 (large effect size), that between MEDS score and mortality was 0.61 (large effect size), and between PIRO score and mortality was 0.28 (medium effect size).

**Table 4:** Comparison of median SPEED, MEDS, and PIRO scores between survivors and non-survivors (n = 127) ^1^Wilcoxon-Mann-Whitney U test SPEED: Sepsis Patient Evaluation in the Emergency Department; MEDS: Mortality in Emergency Department Sepsis; PIRO: Predisposition, Infection, Response, and Organ Dysfunction; IQR: interquartile range

Score	Survivors, median (IQR)	Non-survivors, median (IQR)	P-value
SPEED score	4 (2.5–6)	9 (6.75–11)	<0.001^1^
MEDS score	6 (5–8)	11 (8.75–14)	<0.001^1^
PIRO score	7 (5.5–9)	13 (10.75–14)	<0.001^1^

As shown in Table 5, there was also a significant difference between survivors and non-survivors in terms of distribution of SPEED score quintiles (χ^2^ = 58.469, p <0.001). Using the Cramer's V test, the strength of association between the two variables was 0.68, signifying a high association. Using the bias-corrected Cramer’s V test, the strength of association between the two variables was 0.66, again signifying a high association.

**Table 5:** 28-day mortality rate for SPEED score quintiles (n = 127) SPEED: Sepsis Patient Evaluation in the Emergency Department

SPEED score: category	Outcomes, n (%)			Chi-squared test	
	Survivors	Non-survivors	Total	χ^2^	P-value
<3	21 (33.3%)	1 (1.6%)	22 (17.3%)	58.469	<0.001
4–6	35 (55.6%)	15 (23.4%)	50 (39.4%)		
7–9	6 (9.5%)	22 (34.4%)	28 (22.0%)		
≥10	1 (1.6%)	26 (40.6%)	27 (21.3%)		
Total	63 (100.0%)	64 (100.0%)	127 (100.0%)		

When comparing the individual parameters of the SPEED score between survivors and non-survivors, a significant difference was found between the two groups in the distribution of immunosuppressed state (χ^2^ = 5.752, p = 0.016), hypotension (χ^2^ = 10.226, p = 0.001), hypothermia (χ^2^ = 7.293, p = 0.013), hypoxemia (χ^2^ = 16.773, p <0.001), low hematocrit (χ^2^ = 22.141, p <0.001), elevated blood lactate (χ^2^ = 18.966, p <0.001), and low pH (χ^2^ = 17.390, p <0.001) parameters. However, there was no significant difference between the two groups in terms of distribution of pneumonia (χ^2^ = 1.338, p = 0.247) (Table 6).

Using bias-corrected Cramer’s V test, the strength of the association between immunosuppressed state and mortality was 0.19 (low association), that between hypotension and mortality was 0.27 (low association), between hypothermia and mortality was 0.22 (low association), between hypoxemia and mortality was 0.35 (moderate association), between hematocrit and mortality was 0.41 (moderate association), between blood lactate and mortality was 0.38 (moderate association), and between pH and mortality was 0.36 (moderate association).

**Table 6:** Group comparison of SPEED score parameters between survivors and non-survivors (n = 127) ^1^Chi-squared test. ^2^Fisher’s exact test SPEED: Sepsis Patient Evaluation in the Emergency Department

SPEED score parameters	Survivors, n (%)	Non-survivors, n (%)	P-value
Immunosuppressed state	2 (3.2%)	10 (15.6%)	0.016^1^
Hypotension (systolic blood pressure <90 mmHg)	16 (25.4%)	34 (53.1%)	0.001^1^
Hypothermia (body temperature <36 °C)	0 (0.0%)	7 (10.9%)	0.013^2^
Hypoxemia (pulse oximetry <90%)	28 (44.4%)	51 (79.7%)	<0.001^1^
Low hematocrit (<0.38)	20 (31.7%)	47 (73.4%)	<0.001^1^
Elevated blood lactate (>2.4 mmol/L)	18 (28.6%)	43 (67.2%)	<0.001^1^
Pneumonia	34 (54.0%)	41 (64.1%)	0.247^1^
Low pH (<7.35)	20 (31.7%)	44 (68.8%)	<0.001^1^

**Figure 1:**
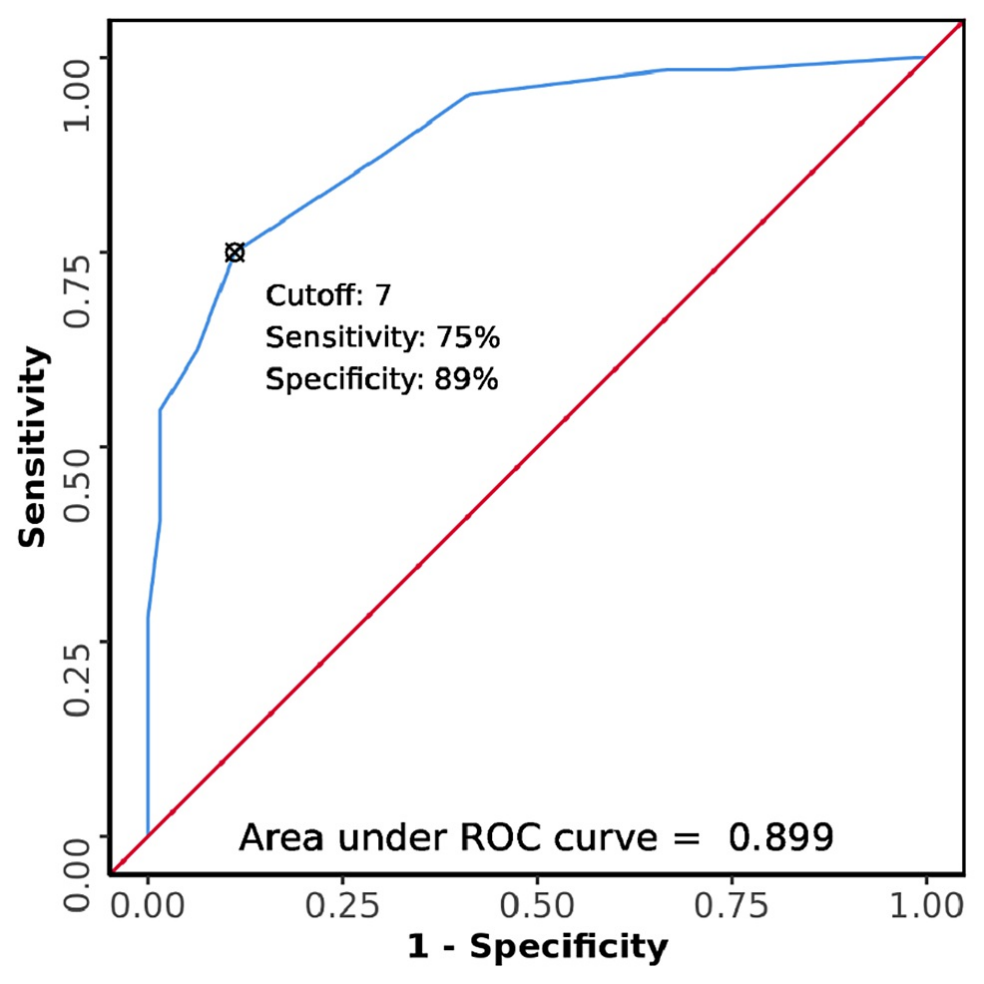
ROC curve of SPEED score for 28-day mortality among patients with sepsis who presented to the emergency department ROC: receiver operating characteristic; SPEED: Sepsis Patient Evaluation in the Emergency Department

The area under the receiver operating characteristic curve (AUROC) for SPEED score for predicting 28-day mortality among patients with sepsis was 0.899 (95% CI: 0.847-0.951), thereby demonstrating good diagnostic performance (Figure 1). It was statistically significant (p <0.001). At a cut-off of ≥7, the SPEED score predicts 28-day mortality with a sensitivity of 75% and a specificity of 89% (Table 7).

**Table 7:** ROC curve analysis showing diagnostic performance of SPEED score, MEDS score, and PIRO score in predicting 28-day mortality among patients with sepsis (n = 127) AUROC: area under the receiver operating characteristic curve; CI: confidence interval; SPEED: Sepsis Patient Evaluation in the Emergency Department; MEDS: Mortality in Emergency Department Sepsis; PIRO: Predisposition, Infection, Response, and Organ Dysfunction

Parameter	SPEED score	MEDS score	PIRO score
	Value (95% CI)	Value (95% CI)	Value (95% CI)
AUROC	0.899 (0.847–0.951)	0.857 (0.793–0.92)	0.895 (0.838–0.951)
Cut-off (p-value)	≥7 (<0.001)	≥10 (<0.001)	≥10 (<0.001)
Sensitivity	75.0% (63–85)	71.9% (59–82)	87.5% (77–94)
Specificity	88.9% (78–95)	84.1% (73–92)	77.8% (66–87)
Positive predictive value	87.3% (76–95)	82.1% (70–91)	80.0% (69–89)
Negative predictive value	77.8% (66–87)	74.6% (63–84)	86.0% (74–94)

**Figure 2:**
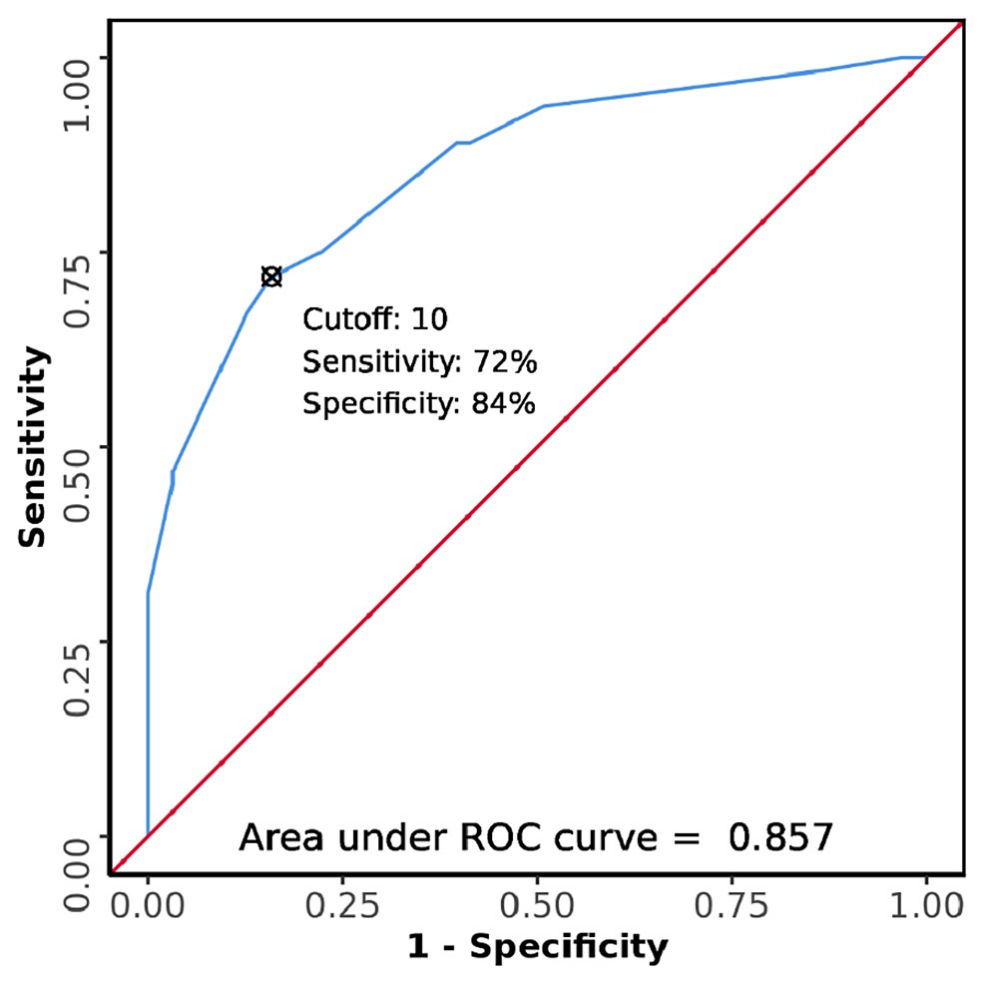
ROC curve of MEDS score for 28-day mortality among patients with sepsis who presented to the emergency department ROC: receiver operating characteristic; MEDS: Mortality in Emergency Department Sepsis

The AUROC for MEDS score for predicting 28-day mortality among patients with sepsis was 0.857 (95% CI: 0.793-0.92), thereby demonstrating good diagnostic performance (Figure 2). It was statistically significant (p <0.001). At a cut-off of ≥10, the MEDS score predicts 28-day mortality with a sensitivity of 72% and a specificity of 84% (Table 7).

**Figure 3:**
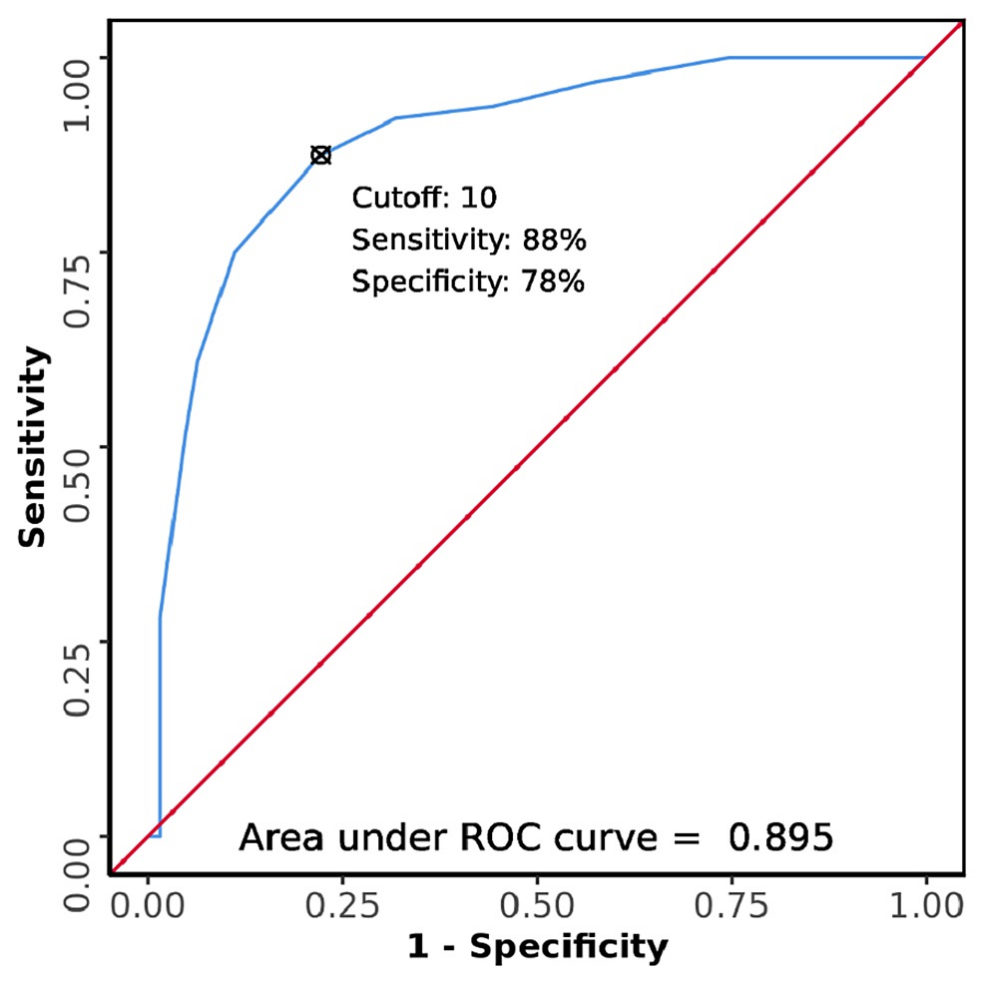
ROC curve of PIRO score for 28-day mortality among patients with sepsis who presented to the emergency department ROC: receiver operating characteristic; PIRO: Predisposition, Infection, Response, and Organ Dysfunction

The AUROC for the PIRO score for predicting 28-day mortality among patients with sepsis was 0.895 (95% CI: 0.838-0.951), thus demonstrating good diagnostic performance (Figure 3). It was statistically significant (p <0.001). At a cut-off of ≥10, the PIRO score predicts 28-day mortality with a sensitivity of 88% and a specificity of 78% (Table 7).

**Figure 4:**
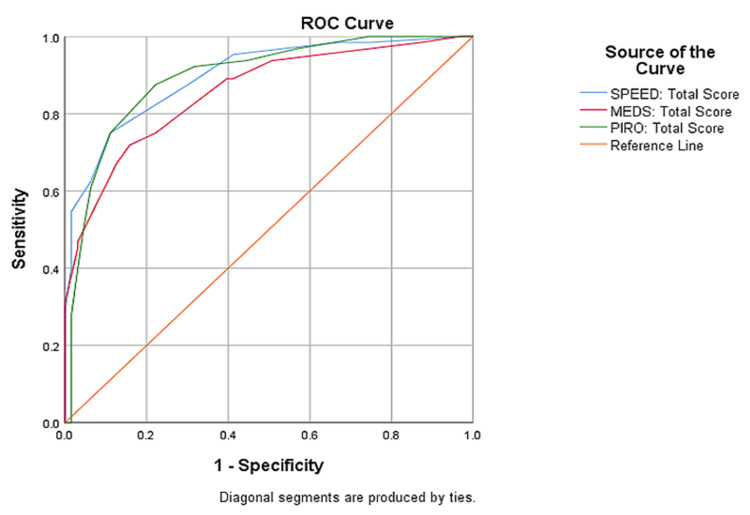
ROC curves of the SPEED score, MEDS score, and PIRO score for 28-day mortality among patients with sepsis who presented to the emergency department ROC: receiver operating characteristic; SPEED: Sepsis Patient Evaluation in the Emergency Department; MEDS: Mortality in Emergency Department Sepsis; PIRO: Predisposition, Infection, Response, and Organ Dysfunction

Figure 4 shows the diagnostic performances of the SPEED, MEDS, and PIRO scores in predicting 28-day mortality among patients with sepsis. There was no significant difference between the diagnostic performance of the SPEED score and MEDS score (DeLong test p = 0.182), nor between the SPEED score and PIRO score (DeLong test p = 0.904). There was also no significant difference in the diagnostic performance between the PIRO score and MEDS score (DeLong test p = 0.203).

## Discussion

In this prospective observational study, the 28-day mortality was found to be 50.4%. Similar to our findings, the study by Todi et al. showed a 28-day mortality rate of 57.6% in patients with severe sepsis [20]. The study by Darba and Marsà reported similar findings, with a case fatality rate of 42.9% in patients with septic shock [21]. In the study by Chatterjee et al., the 28-day mortality rate of patients with severe sepsis was reported to be 62.8%, which is considerably higher than in the current study [22]. This difference can be attributed to their study design: sepsis was defined in their study as the presence of both infection and systemic inflammatory response syndrome (SIRS), while severe sepsis was defined as the presence of sepsis fulfilling at least one criterion for organ dysfunction. This study included patients with sepsis as defined by the qSOFA criteria. Thus, the study by Chatterjee et al. included a much sicker population. Pneumonia was the most common site of infection in our study, followed by urinary tract infections. The respiratory tract has been reported as the most common site of infection among patients with sepsis in several previous studies [22-26].

All the three scores assessed in the study (SPEED, MEDS, and PIRO) were found to be a significant predictor of 28-day mortality among patients with sepsis (p <0.001). The mortality rate was higher in patients with a higher SPEED score, as shown in Table 5. These findings are similar to those by Bewersdorf et al. and Elbaih et al. [17,27]. The SPEED score is a composite score comprising eight parameters: immunosuppressed state, hypotension, hypothermia, hypoxemia, low hematocrit, pneumonia, elevated blood lactate, and acidosis. Among these eight parameters, all except pneumonia were found to be significantly associated with mortality. Multiple studies conducted previously have reported that the presence of an immunosuppressed state, hypotension, hypothermia, hypoxemia, low hematocrit, elevated blood lactate, and acidosis were significantly associated with mortality among patients with sepsis [28-44]. Pneumonia was not found to be significantly associated with mortality among patients with sepsis in this study (p = 0.247). Similar findings have been reported in the studies by Xie et al., Huang et al., and Wang et al. [24,25,28]. Bewersdorf et al. have reported that all eight parameters of the SPEED score were significantly associated with mortality [17]. However, only two parameters - hypotension and hypoxemia - were reported as significantly associated with mortality by Elbaih et al. [27].

The AUROC for the SPEED score in this study was 0.899 (95% CI: 0.847-0.951). The cut-off value of the SPEED score obtained was ≥7. At this value, the SPEED score predicted mortality with a sensitivity of 75%, specificity of 89%, a positive predictive value of 87.3%, and a negative predictive value of 77.8%. In the study by Bewersdorf et al., the AUROC for the SPEED score was 0.83 (95% CI: 0.79-0.88) in the derivation set and 0.80 (95% CI: 0.73-0.87) in the validation set [17]. In the study by Elbaih et al., the AUROC for SPEED score was 0.87 (95% CI: 0.788-0.963) [27]. Although none of these studies reported a cut-off value for the SPEED score, the AUROC in these studies is quite similar to the one obtained in the current study.

The AUROC for the MEDS score was 0.857 (95% CI: 0.793-0.92). The cut-off value of the MEDS score obtained was ≥10. At this value, the MEDS score predicted mortality with a sensitivity of 71.9%, specificity of 84.1%, a positive predictive value of 82.1%, and a negative predictive value of 74.6%. In the study by Shapiro et al., the AUROC for the MEDS score for predicting mortality in the derivation set was 0.82, while it was 0.78 in the validation set [13]. In a meta-analysis by Zhang et al., the AUROC for the MEDS score was 0.83 (95% CI: 0.80-0.86), with a pooled sensitivity of 79% and specificity of 74% to predict mortality. A substantial variation was found in the cut-off value of the MEDS score used to predict mortality, with values ranging from 7 to 14.5. However, MEDS scores of 8-12 were most frequently chosen as the optimal cut-off values, with 8 being the most frequent value [45].

The value of the AUROC obtained in this study was found to be quite similar to the above-mentioned studies. The cut-off score obtained in this study was 10, which is higher than what was reported in the meta-analysis by Zhang et al. This was due to choosing a value that had a higher specificity as compared to sensitivity. The hospital care center in this study receives extremely ill patients, and hence specificity is of greater importance than sensitivity in this setting.

The AUROC for the PIRO score was 0.895 (95% CI: 0.838-0.951) in this study, with an obtained cut-off value of ≥10. At this value, the PIRO score predicted mortality with a sensitivity of 87.5%, specificity of 77.8%, a positive predictive value of 80.0%, and a negative predictive value of 86.0%. The value of AUROC obtained is quite similar to that by Rathour et al. In their study, the AUROC of the PIRO score for predicting mortality was reported as 0.94 (95% CI: 0.900-0.971) [12]. However, the study by Caramello et al. reported the AUROC for PIRO score as 0.765 (95% CI: 0.71-0.82) for 30-day mortality and 0.754 (0.701-0.806) for 60-day mortality [46]. None of these studies reported a cut-off value for the PIRO score. In the current study, the SPEED score was found to be the best predictor of 28-day mortality among patients with sepsis, with an AUROC of 0.899. This was followed by the PIRO score with an AUROC of 0.895, and the MEDS score with an AUROC of 0.857. No statistically significant difference was found between the diagnostic performance of the three scores. Similar findings have been reported in the studies by Bewersdorf et al. and Elbaih et al. [17,27].

The MEDS score is used widely in the ED for risk stratification of patients with sepsis. The PIRO score is a detailed classification system involving multiple parameters, which was designed for use in the ED. Both MEDS and PIRO scores require the band cell percentage for calculation, which may not be available to ED physicians everywhere. The PIRO score additionally requires activated partial thromboplastin time (aPTT) values for calculation, which again may not be readily available to ED physicians. The SPEED score is a much simpler score, and it utilizes parameters that are readily available even in resource-limited settings. Since the diagnostic performances of all these scores are quite similar, the SPEED score has the advantage of being the simple and easy choice to reliably predict mortality.

Limitations

This study has some limitations. Firstly, the sample size was small; hence, a larger, multi-center study would be required before applying these results to the general population. Secondly, very few patients satisfied certain parameters of the individual scores, such as hypothermia and nursing home residency. Thus, the relevance of these individual parameters in predicting mortality among patients with sepsis could not be accurately determined. Thirdly, the likely infective agents responsible for sepsis in this study’s healthcare setting may be different from other healthcare settings, and this could confound the utility of the scores. Additionally, death due to causes other than infection could not be entirely excluded. Finally, these findings cannot be extrapolated to children and pregnant females.

## Conclusions

The SPEED score is a simple and handy tool that relies on parameters readily available at the point-of-care in the ED, and it is also suitable for usage in resource-limited settings. In this study, there was no significant difference between the diagnostic performances of the SPEED, MEDS, and PIRO scores in predicting the 28-day mortality among patients with sepsis. Hence, the SPEED score can be utilized as one of the early risk stratification methods for patients with sepsis in the ED. This can help in allocating healthcare resources rationally in terms of timely admission of patients to an appropriate level of care, ultimately resulting in a significant impact on patient outcomes.
